# Supplementary material for: WordCommentsAnalyzer: A windows software tool for qualitative research
Source: F1000Res. 2018 Sep 4;7:536. Originally published 2018 May 3. [Version 2] doi: 10.12688/f1000research.14819.2 (PMC6137411; doi:10.12688/f1000research.14819.2)
Supplement: Supplementary file 3 [file f1000research-7-17614-s0002.tgz › b2a79496-4ada-47c4-9682-a99ce867836d.zip!/Eight Iranian Health Journals/The Journal of Toloo-e-Behdasht_24.docx]

© The Journal of Toloo-e-Behdasht. This work is licensed under CC BY 4.0 (https://creativecommons.org/licenses/by/4.0/).

Volume & Issue: دوره 13 - شماره 3

Title: بررسی عوامل موثر برکارایی نانو ذرات روی اکسید در حذف کادمیوم از محلول‌های آبی

http://tbj.ssu.ac.ir/article-1-1113-fa.html

مقدمه: کادمیوم یک فلز سنگین با سمیت بسیار بالا می‌باشد که به عنوان ماده‌ای سرطانزا شناخته شده است. بنابراین لازم است که برای حفظ سلامت انسان‌ها و حفاظت از محیط زیست یون کادمیوم اضافی در پساب های صنعتی قبل از تخلیه آن به آب سطحی و زیرزمینی حذف گردد. لذا در این مطالعه کارایی نانو ذرات روی اکسید در حذف کادمیوم از محلول‌های آبی مورد بررسی قرار گرفته است. روش بررسی: این تحقیق یک مطالعه تجربی- آزمایشگاهی می‌باشد. آزمایشات جذب به صورت ناپیوسته در مقیاس آزمایشگاهی با استفاده از نانو ذرات روی اکسید انجام شد و اثر غلظت اولیه کادمیوم، مقدار جاذب، pH و زمان تماس بر روی راندمان حذف بررسی شد. مقدار کادمیوم باقی مانده با استفاده از دستگاه جذب اتمی Varian AA20 ساخت کشور استرالیا اندازه گیری شد و برای رسم نمودارها از نرم افزار Excel استفاده گردید. یافته‌ها: در این مطالعه نتایج نشان داد که زمان تماس بهینه برای حذف یون کادمیوم 15 دقیقه بدست آمد و با افزایش جرم جاذب و زمان تماس راندمان حذف کادمیوم افزایش و با افزایش غلظت اولیه کادمیوم از 5 به 25 میلی گرم در لیتر راندمان حذف از 6/86 به 5/60 کاهش می یابد. همچنین مشخص گردید که با افزایش غلظت اولیه کادمیوم میزان جذب افزایش می‌یابد. حداکثر کارایی جذب در pH برابر با 7 به میزان 6/89% بدست آمد. نتیجه گیری: با توجه به نتایج بدست آمده نانو ذرات روی اکسید کارایی مناسبی در حذف کادمیوم از محیط‌های آبی دارد. و می‌تواند در تصفیه فاضلاب حاوی یون کادمیوم استفاده شود. هر چندکه عملکرد آن بستگی زیادی به قدرت یونی و تداخل دیگر فلزات موجود در فاضلاب دارد.

Title: تاثیر مداخلات مهندسی و مدیریتی بر ارتقاء سطح شاخص های فرهنگ ایمنی در کارگران یک کارخانه لوله نورد سازی

http://tbj.ssu.ac.ir/article-1-1112-fa.html

مقدمه: سازمان تأمین اجتماعی کشور تعداد حوادث ناشی از کار را در شش ماه اول سال 1388، 9 هزار و 856 مورد تخمین زده است که بخش عمده ای از این حوادث در یک سیستم مدیریت ایمنی نادرست همراه با سطح پایین شاخص های فرهنگ ایمنی شکل گرفته است. لذا مطالعه حاضر در جهت ارتقاء سطح شاخص های فرهنگ ایمنی مبتنی بر اجرای مداخلات مهندسی و مدیریتی در کارگران یک کارخانه لوله نورد سازی در شهر همدان در سال های 1389 و 1390 صورت پذیرفت. روش بررسی: در یک مطالعه توصیفی- تحلیلی به روش قبل و بعد، بر روی کلیه کارگران یک کارخانه لوله نورد سازی در شهر همدان (120 نفر) به منظور بررسی سطح شاخص های فرهنگ ایمنی، قبل و بعد از انجام مداخلات مهندسی و مدیریتی از نسخه فارسی پرسشنامه استاندارد (Health and Safety Executive) HSE استفاده گردید. یافته ها: نتایج حاصل از این تحقیق نشان می دهند میانگین سطح شاخص نهایی فرهنگ ایمنی کارگران مورد مطالعه قبل از انجام مداخلات مهندسی و مدیریتی از 204 با انحراف معیار 42/43 (فرهنگ ایمنی منفی) به 11/31 ± 318 (فرهنگ ایمنی مثبت) پس از انجام مداخلات ارتقاء یافته است. همچنین نتایج نشان می دهند تمامی شاخص های دهگانه فرهنگ ایمنی نیز پس از انجام مداخلات به میزان قابل توجهی افزایش یافته اند (001/0P<). نتیجه گیری: نتایج مطالعات گوناگون نشان می دهند اولویت اولیه جهت خلق فرهنگ ایمنی مثبت، آموزش می باشد که این مهم پس از انجام مداخلات در کارخانه مذکور میسر و واحد آموزش تاسیس و شرح وظایف آن مشخص گردید. نتایج حاصل از مطالعه حاضر نشان می دهند که با افزایش سن و سابقه کار، سطح شاخص نهایی فرهنگ ایمنی کارگران افزایش یافته است.

Title: بررسی آلودگی باکتریایی بستنی های سنتی و پاستوریزه شهر اراک در تابستان و پائیز 1390

http://tbj.ssu.ac.ir/article-1-1115-fa.html

مقدمه: بستنی از جمله فرآورده های لبنی است که به دلیل دارا بودن ترکیبات مغذی، محیطی مناسب جهت فعالیت انواع میکروارگانیسم ها می باشدکه در صورت عدم رعایت بهداشت در مراحل تولید، نگهداری و عرضه می تواند باعث بروز مسمومیت و ایجاد بیماری های روده ای در بین مصرف کنندگان گردد. هدف از انجام این مطالعه بررسی آلودگی باکتریایی در بستنی های سنتی ( غیر پاستوریزه) و بستنی های پاستوریزه در شهر اراک بود. روش بررسی: نمونه های مورد مطالعه از مراکز عرضه این محصول در سطح شهر اراک با استفاده از روش نمونه گیری خوشه ای تک مرحله ای جمع آوری گردید. شهر اراک به 5 بخش تقسیم شده و هر بخش به عنوان یک خوشه محسوب و سپس بر اساس میزان تراکم عرضه بستنی در هر خوشه تعداد نمونه ها بطور تصادفی انتخاب گردید که مجموعا 110 نمونه بوده و شامل 70 نمونه بستنی غیرپاستوریزه(سنتی) و 40 نمونه بستنی پاستوریزه که از تیر تا آذرماه 1390 به مدت 6 ماه تهیه و در شرایط استریل با حفظ زنجیره سرد به آزمایشگاه میکروبیولوژی منتقل شده و طبق استاندارد ملی ایران آزمونهای استافیلوکوکوس آرئوس کواگلاز مثبت با استفاده از محیط کشت برد پارکر آگار و کلی فرم ها با محیط کشت ویولت رد بایل آگار و برای جداسازی سالمونلا از محیط های کشت سالمونلا شیگلا آگار، بریلیانت گرین آگار و بیسموت سولفیت آگار و در نهایت انجام تستهای تائیدی انجام پذیرفت. یافته ها: نتایج این بررسی نشان داد 14/57 درصد(40 نمونه) از نمونه های غیر پاستوریزه، غیرقابل مصرف و باکتریهای موجود در آنها عبارت بودند از: استافیلوکوکوس اورئوس کواگلاز مثبت (50%)، گونه های کلبسیلا(28/54%)، اشریشیا کلی(85/42%). در بستنی های پاستوریزه، 30 درصد(12 نمونه ) از نمونه ها غیر قابل مصرف بودند و باکتریهای موجود در آنها عبارت بودند از: اشریشیا کلی(25%)، استافیلوکوک اورئوس کواگلاز مثبت (10%)، کلبسیلا نومونیه(20%). تمامی نمونه ها از نظر سالمونلا منفی بوده اند. نتیجه گیری: نتایج این بررسی نمایانگر وضعیت نامطلوب بستنی های سنتی و پاستوریزه شهر اراک از نظر آلودگی باکتریایی می باشد.

Title: رابطه شوخ طبعی و کیفیت زندگی در بیماران سرطانی

http://tbj.ssu.ac.ir/article-1-1117-fa.html

مقدمه: شوخ طبعی به عنوان سلاح مقابله با مشکلات و نیرویی شفابخش، توجهات بسیار جدی را به خود جلب کرده است. از آن جایی که بیماری‌های جسمانی نظیر سرطان می‌تواند بر کیفیت زندگی که شامل عمل‌کرد مطلوب در ابعاد جسمانی و روان‌شناختی می‌باشد، اثرات منفی داشته باشد. هدف این پژوهش، بررسی نقش ابعاد شوخ طبعی بر ابعاد کیفیت زندگی بیماران سرطانی بوده است. روش بررسی: روش پژوهش، توصیفی و از نوع همبستگی بوده است که بر روی 50 نفر از بیماران سرطانی بیمارستان علی بن ابیطالب شهر زاهدان به صورت نمونه‌گیری در دسترس صورت گرفت و پرسش نامه‌های کیفیت زندگی (36SF-) و شوخ طبعی (SHQ) در مورد آنها اجرا گردید. داده‌ها با استفاده از ضریب همبستگی پیرسون و تحلیل رگرسیون تجزیه و تحلیل شد. یافته‌ها: نتایج نشان داد که بین ابعاد شوخ طبعی (شوخ طبعی در شرایط استرس آور، روابط اجتماعی، شوخی کلامی و خنده) با کیفیت زندگی جسمانی(025/0 P≤، 278/0=r ) و کیفیت زندگی روانی (023/0 P≤، 283/0=r ) رابطه مثبت و معنی داری وجود دارد. نتایج رگرسیون نشان داد که شوخ طبعی در روابط اجتماعی به همراه شوخی کلامی 24/0 از تغییرات مربوط به کیفیت زندگی جسمانی و این دو متغیر با هم 40/0 از تغییرات مربوط به کیفیت زندگی روانی را نیز پیش بینی می کنند. نتیجه گیری: با توجه به نقش شوخ طبعی در کیفیت زندگی بیماران سرطانی پیشنهاد می‌شود مداخله‌های لازم جهت افزایش شوخ طبعی در زندگی بیماران سرطانی صورت گیرد.

Title: بررسی توانایی انجام فعالیتهای روزانه و شیوع بیماریهای مزمن در سالمندان شهر یزد

http://tbj.ssu.ac.ir/article-1-1114-fa.html

مقدمه : دردوران پیری و کهولت تغییرات جسمی ‌و روحی بسیاری به صورت تدریجی در انسان ایجاد می‌شودکه افراد را در معرض تهدیدات بالقوه ای نظیر افزایش ابتلا به بیماریهای مزمن، تنهایی و انزوا،عدم برخورداری از حمایت اجتماعی قرار داده و بدلیل ناتوانی های جسمی و ذهنی در موارد زیادی استقلال فردیشان نیز مورد تهدید قرار می گیرد. بررسی مشکلات سالمندان به منظور مدیریت و برنامه ریزی برای این قشر جامعه از اولویت های بهداشتی کشور می باشد. لذا این مطالعه با هدف تعیین وضعیت توانایی انجام فعالیتهای روزانه وشیوع بیماریهای مزمن ، در سالمندان شهریزد انجام شده است. روش بررسی : این مطالعه توصیفی-مقطعی بر روی265 سالمند 60 ساله و بالاتر، ساکن شهر یزد به روش نمونه گیری خوشه ای انجام شد. داده ها با استفاده ازپرسشنامه ای مشتمل بر سوالات دموگرافیک و ابتلا به بیماریهای مزمن و "ابزاراستقلال فعالیت های زندگی روزانه" استفاده شد. داده های جمع آوری شده با استفاده از نرم افزار16spss و جداول توصیفی،تست های کروسکال والیس ومن ویتنی مورد تجزیه و تحلیل قرار گرفت. یافته ها: میانگین نمره کسب شده در فعالیتهای روزمره زندگی99/5± 03/ 36 (40-0)و فعالیتهای ابزاری زندگی برابر با 22/8±25/24 (40- 0) بود به طوریکه 2/56% سالمندان در گروه مستقل و 34% در گروه مستقل نسبی و 8/6% در گروه وابسته نسبی قرار داشتند.همچنین فعالیتهای روزمره زندگی با متغیر های سن(361/0r=- و 001/0 p=)و سطح تحصیلات( 335/0r=، 001/0 p=) ارتباط معنی دار آماری داشت. همبستگی معکوس و معنی داری(332/0- r= 001/0 (p=بین تعداد بیماریهای مزمن و توانایی انجام فعالیتهای روزانه زندگی دیده شد بطوریکه با افزایش تعداد بیماریهای مزمن سطح استقلال سالمندان کاهش یافته بود .همچنین در این بررسی با توجه به اظهارنظر خود سالمندان بیشترین درصد فراوانی بیماریهای مزمن به ترتیب شامل بیماری درد های مفصلی (8/74%)پوکی استخوان (8/53%) فشارخون بالا(4/49%)مشکلات خواب (2/39%) و بیماریهای قلبی عروقی (5/38%) بود. نتیجه گیری: بخش قابل توجهی از سالمندان در جامعه مورد بررسی از نظر فعالیتهای روزانه وابسته نسبی می باشند که در این زمینه توانایی استحمام یکی از مهمترین وابستگی ها می باشد، به علاوه با توجه به نتایج مطالعه، بیماریهای مزمنی چون درد مفاصل، پوکی استخوان از شیوع بالایی برخوردار بوده لذا پیشنهاد می شود در جهت کاهش ناتوانی سالمندان با همکاری مراکز بهداشتی درمانی وشناسایی سالمندان بیمار با درمان به موقع وآموزش خود مراقبتی به سالمند وخانواده اش بتوان تا حدی از بروز معلولیت ها وناتوانی آنها پیشگیری نمود.

Title: بررسی کیفیت میکروبی دستگاه های برش دهنده کالباس در فروشگاه های عرضه مواد غذایی شهر شیراز

http://tbj.ssu.ac.ir/article-1-1116-fa.html

مقدمه: پژوهش حاضر با هدف بررسی کیفیت میکروبی تیغه دستگاه های برش دهنده کالباس در فروشگاه های عرضه مواد غذایی شهر شیراز انجام شد. روش بررسی:در این مطالعه 60 عدد از تیغه های دستگاه های برش دهنده کالباس مربوط به فروشگاههای عرضه مواد غذایی واقع در شمال، جنوب، شرق و غرب شهر شیراز مطابق با استاندارد ایزو نمونه برداری گردید. سپس نمونه ها از نظر ویژگی های میکروبی با استفاده از محیط های کشت اختصاصی آزمایش شدند. داده های حاصل به کمک نرم افزار16 SPSS و آزمونهای آمار توصیفی و ANOVA مورد تجزیه و تحلیل قرار گرفت. یافته ها: از نظر شمارش کلی باکتریایی، کلی فرم و استافیلوکوکوس اورئوس کواگولاز به ترتیب 23، 4 و 6 درصد از تیغه دستگاههای برش دهنده کالباس وضعیت غیر رضایت بخشی داشتند. تنها در دو مورد استافیلوکوکوس اورئوس کواگولاز مثبت در سطوح تیغه مشاهده گردید. همه تیغه های برش دهنده عاری از سالمونلا و اشریشیاکلی بودند. کمترین میزان شمارش کلی باکتریایی، کلی فرم و استافیلوکوکوس اورئوس به ترتیب مربوط به شمال، غرب و جنوب شهر شیراز بود. نتیجه گیری: نتایج این مطالعه نشان داد که آلودگی برخی از دستگاههای برش دهنده کالباس به دلایلی مانند روش شستشوی نامناسب و دستکاری های غیر بهداشتی بیش از حد استاندارد است. بر اساس داده های بدست آمده تلاش بیشتری در راستای بکار گیری اصول بهداشت فردی و بهداشت محیط در مراکز عرضه مواد غذایی مورد نیاز می باشد.

Title: استرس شغلی در کارکنان نوبت‌کار و غیرنوبت‌کار یکی از صنایع لبنی یزد

http://tbj.ssu.ac.ir/article-1-1118-fa.html

مقدمه: بسیاری از صنایع نیازمند کار به صورت شبانه‌روزی و ارائه خدمت به طور تمام وقت هستند. بنابراین، افراد و سازمان‌ها با مسائل جدیدی از جمله نوبت‌کاری مواجه هستند. ضرورت توجه به محیط کار و سلامت و استرس شغلی کارکنان در صنایع، منجر به انجام پژوهشی با هدف بررسی ارتباط بین استرس شغلی و نوبت‌کاری در کارکنان یکی از صنایع لبنی یزد گردید. روش بررسی: در یک پژوهش توصیفی- تحلیلی و از نوع مقطعی، استرس شغلی 100 نفر از کارکنان یکی از مجتمع‌های صنعتی که از میان جامعه (1000 نفر) به طور تصادفی انتخاب شدند، با استفاده از ‌پرسشنامه‌ استرس شغلی مورد مطالعه قرارگرفت. به منظور آزمون فرضیه‌های پژوهش از همبستگی، آزمون t مستقل و تحلیل رگرسیون چند متغیری (بطور همزمان) استفاده گردید. یافته‌ها: نتایج نشان می دهد که استرس ‌شغلی در نوبت‌کاران بیشتر از کارکنان غیرنوبت‌کار بود. به علاوه، بین میزان (99/ 8F= و 01/0α=) و منابع استرس شغلی کارکنان (76/3F= و 05/0α=) و متغیرهای جمعیت‌شناختی و نیز بین تحصیلات و استرس کارکنان ارتباط معناداری مشاهده شد. این پژوهش هم چنین نشان‌داد که، استرس را می‌توان از طریق متغیرهای جمعیت‌شناختی پیش‌بینی نمود (35/0F= و 05/0α=) و بین میزان (78/0 =r) و منابع استرس شغلی (54/0 =r) و نوبت‌کاری همبستگی مثبت و معناداری وجود دارد. نتیجه‌گیری: بین دو متغیر استرس‌شغلی و نوبت‌کاری ارتباط معناداری وجود دارد. از این رو انجام چنین پژوهش‌هایی توصیه می شود؛ چراکه از نتایج آنها می‌توان به راهکارهای عملیاتی و مداخلات طراحی شغل مربوط به نوع کار و ارگونومی محیط کار برای بهبود استرس شغلی و سلامت کارکنان دست‌یافت.

Title: تأثیر آموزش مدیریت خشم بر افزایش سلامت روان مادران دارای فرزند معلول

http://tbj.ssu.ac.ir/article-1-1119-fa.html

مقدمه: فرایند تولد کودک برای والدین لذت‌آور است گرچه این فرایند با مشقات فراوانی همراه است. امید به سالم بودن کودک معمولاً احساس اعتماد در والدین ایجاد می‌کند و کودک خود را می پذیرند، ولی به محض آگاهی آنها از معلولیت فرزند خود، تمام امیدها به یأس مبدل می‌گردد و مشکلات شروع می‌شود. استرس، افسردگی و خشم از جمله هیجانات رایجی هستند که این والدین تجربه می‌کنند. هدف کلی این تحقیق بررسی تأثیر آموزش مدیریت خشم بر افزایش سلامت روان مادران دارای فرزند معلول در شهر یزد می‌باشد. روش بررسی: پژوهش حاضر از نوع تحقیقات آزمایشی با طرح پیش آزمون ـ پس آزمون با گروه گواه است. جامعه آماری شامل کلیه مادران دارای فرزند معلول تحت پوشش بهزیستی شهرستان یزد در سال 91- 1390 می‌باشد. نمونه مورد نظر شامل 35 مادر است که به صورت تصادفی در دو گروه آزمایش و کنترل قرار گرفته‌اند (18 نفر گروه آزمایش و 17 نفر گروه گواه). از پرسشنامه سلامت روان برای گردآوری داده ها استفاده شد. برنامه آموزش مدیریت خشم، بر اساس رویکرد شناختی ـ رفتاری به مدت 7 جلسه 2 ساعته بر روی گروه آزمایش اعمال شد. داده‌ها با استفاده از شاخص‌های آمار توصیفی (میانگین و انحراف معیار) و آزمونt مستقل بر روی تفاضل نمرات پیش آزمون – پس آزمون مورد تحلیل قرار‌گرفت. یافته ها: یافته ها نشان داد که آموزش مدیریت خشم به تفاوت معنادار آماری (05/0>p) بین مراحل قبل و پس از آزمون در نمره کلی سلامت روان و زیرمقیاس های علایم جسمانی، اضطراب و افسردگی (به استثنای اختلال در کارکرد اجتماعی) می‌انجامد. همچنین آموزش مدیریت خشم بر کاهش نشانه‌های جسمانی و همچنین کاهش افسردگی مادران جوان در مقایسه با مادران میانسال تأثیر بیشتری دارد. نتیجه گیری: با توجه به ثمربخش بودن استفاده از برنامه آموزشی مدیریت خشم در افزایش سلامت روان، آموزش این برنامه در راستای کاهش فشار روانی باید صورت گیرد.

Title: بررسی تأثیر گازسوزشدن کوره های آجرپزی استان یزد در کاهش انتشار آلاینده های هوا

http://tbj.ssu.ac.ir/article-1-1120-fa.html

مقدمه: جایگزینی نفت کوره با گاز طبیعی در کوره های آجرپزی، یکی از عوامل مؤثر بر کاهش آلودگی هوا و همچنین کاهش هزینه این کوره ها است. هدف از انجام این مطالعه، برآورد میزان کاهش آلاینده های هوا در نتیجه گازسوزشدن کوره های آجرپزی استان یزد می باشد. روش بررسی: در این تحقیق توصیفی، میزان مصرف گاز طبیعی 46 کوره آجرپزی استان یزد از ابتدای گازسوزشدن آنها تا پایان شهریور 91 محاسبه و میزان نفت کوره معادل آن بر اساس ارزش حرارتی آنها برآورد شد. سپس آلاینده های هوای تولیدی ناشی از مصرف گاز طبیعی و نفت کوره در این مدت با هم مقایسه و میزان کاهش هر کدام از آلاینده ها بر حسب تن مشخص شد. در نهایت میزان صرفه جویی ریالی و صرفه جویی در هزینه های اجتماعی تخریب محیط زیست ناشی از استفاده از گاز طبیعی به جای نفت کوره محاسبه شد. یافته ها: نتایج این تحقیق نشان داد که از ابتدای گازسوزشدن کوره های آجرپزی استان یزد (از سال 87) تا پایان شهریور 91، از تولید و انتشار گاز CO2 به میزان 71/130485 تن و انتشار سایر آلاینده ها به میزان 63/8695 تن به اتمسفر کاسته شده است. میزان صرفه جویی ریالی ناشی از گازسوزشدن این کوره ها در طی این مدت، 578/187 میلیارد ریال بوده است . علاوه بر صرفه جویی ریالی ذکرشده، مقدار380/129 میلیارد ریال در هزینه های اجتماعی و زیست محیطی ناشی از انتشار آلاینده ها به اتمسفرصرفه جویی شده است. نتیجه گیری: با توجه به آثار مثبت اقتصادی، زیست محیطی و بهداشتی ناشی از گازسوزشدن کوره های آجرپزی، برنامه ریزی و تأمین اعتبار جهت گازسوزنمودن سایر کوره های آجرپزی ضروری است.

Title: تحلیل مشتری مرتبط با مصرف ماهی، موانع مصرف و عوامل موثر بر آن با استفاده از چهارچوب بازاریابی اجتماعی درمیان زنان شهر یزد در سال 1391

http://tbj.ssu.ac.ir/article-1-1121-fa.html

مقدمه:سازمان جهانی بهداشت مصرف ماهی به میزان دو بار در هفته را توصیه می کند، اما با این حال مطالعات و گزارش ها نشان می دهد میزان مصرف ماهی دربرخی از کشورها کمتر از این میزان است. هدف این مطالعه تحلیل مشتری مرتبط با مصرف ماهی، موانع و عوامل موثر بر آن با استفاده از چهارچوب بازاریابی اجتماعی درمیان زنان شهر یزد و شناسایی اقدامات مداخله ای موثر بر افزایش مصرف ماهی از دیدگاه زنان شهر یزد است. روش بررسی: مطالعه از نوع کیفی بود که چهار مرحله اول ابزار بازاریابی اجتماعی شامل برنامه ریزی مقدماتی، تحلیل مشتری، تحلیل ابعاد چهار گانه بازار و نیز تحلیل کانال را شامل می شد. پس از برگزاری سه جلسه بحث گروهی متمرکز با حضور 36 نفر از زنان خانه دار مراجعه کننده به مراکز بهداشتی و درمانی شهر یزد و نیز سه جلسه بحث گروهی متمرکز با 24 نفر از زنان شاغل در ادارات آموزش و پرورش، شهرداری و دانشگاه علوم پزشکی یزد بصورت جداگانه، دیدگاهها، خواستها و ترجیحات آنان درباره مصرف ماهی و عوامل موثر بر آن یا موانع مصرف و روشهای موثر ترویج ماهی استخراج و دسته بندی شد. یافته ها: زنان خانه دار و شاغل به موانع مشابه و گاهی نیز متفاوتی برای مصرف ماهی و نیز عوامل موثر بر مصرف اشاره نمودند. این موانع شناسایی شده، از بعد قیمت شامل قیمت بالای ماهی، ناخوشایند بودن طعم ماهی، داشتن تیغ و بوی نامطبوع و از بعد عوامل مرتبط با مشتری شامل عدم علاقه یکی از اعضای خانواده بود. از نظر زنان شاغل، عدم آشنایی با نحوه پخت (بعدترویج)، قیمت بالای ماهی، بوی نامطبوع و طعم بد (بعد قیمت)، طبع و مزاج مردم، عدم علاقه یکی از اعضای خانواده و کمبود وقت (بعدعوامل مرتبط با مشتری) مهمترین موانع مصرف بیان شد. نتیجه گیری: دیدگاهها، خواستها و ترجیحات افراد نسبت به مسائل و نیز محصولات مختلف از جمله ارتباط با سلامتی متاثر از شرایط فردی و محیطی شامل شرایط بیولوژیک و سطح سلامت، شرایط فرهنگی، اجتماعی، سیاسی، اقتصادی و زیست محیطی است که با گذر زمان و نیز تغییر این شرایط می تواند تغییر کند. به همین دلیل است که مفاهیم چهارچوب بازاریابی تاکید بر شناخت کامل و به روز از مشتریان هر محصول و نیز طبقه بندی نمودن مشتریان و تفاوتهای ایشان در طراحی و اجرای برنامه های بازاریابی اجتماعی دارد تا اطمینان حاصل شود برنامه های مداخله ای موفق به دسترسی به انواع و تعداد بیشتری از مشتریان شده و باعث ایجاد تغییر مورد نظر جهت مصرف آن محصول در ایشان شود.

Title: بررسی سرواپیدمیولوژی عفونت توکسوپلاسمادر زنان باردار شهر یزد سال 1391

http://tbj.ssu.ac.ir/article-1-1122-fa.html

مقدمه: توکسوپلاسموز یکی از شایع ترین عفونتهای انگلی مشترک انسان و دام در سراسر دنیاست که توسط تک یاخته ای به نام توکسوپلاسما گوندی (Toxoplasma gondii) ایجاد می شود. ابتلا مادران باردار به این عفونت خصوصا در سه ماهه اول بارداری می تواند منجر به سقط جنین و یا تولد نوزاد با عوارض سیستم عصبی و چشمی شود. انجام اینگونه مطالعات می تواتد راهکارهای مناسبی برای درمان و پیشگیری از بروز این عوارض فراهم نماید. روش بررسی: در این مطالعه توصیفی مقطعی به منظور تعیین میزان آنتی بادی اختصاصی ضد توکسوپلاسما گوندی در زنان باردار شهر یزد در سال1392، تعداد 181 نمونه سرم از مراجعین به مراکز بهداشتی - درمانی شهری جهت انجام معاینات بارداری به روش تصادفی ساده تهیه و با روش ELISA مورد آزمایش قرار گرفت. داده ها با آزمونهای آماری از جمله chi-square تجزیه و تحلیل گردید. یافته ها: در این مطالعه، از 181 خانم باردار تعداد 58 نفر (32 %) از نظر آنتی بادی IgG ضد توکسوپلاسما سرم مثبت بودند. بر اساس نتایج ، بین سطح عیار IgG و سن افراد (001/0 (p<و همچنین بین سطح عیار آنتی بادی IgG و سطح تحصیلات افراد رابطه آماری معنی دار مشاهده گردید( 005/0. (p<، اما بین عیار مثبت آنتی بادی و سایر متغیرها ارتباط معنی داری دیده نشد. نتیجه گیری: بیش از نیمی از جمعیت مورد مطالعه از نظر وجود آنتی بادی ضد توکسوپلاسما سرم منفی و حساس به عفونت می باشند. لذا انجام مراقبتهای سرولوژیک از نظر توکسوپلاسما در دوران بارداری و نیز ارائه آموزشهای لازم جهت ارتقاء آگاهی نسبت به راههای پیشگیری از توکسوپلاسموز مادرزادی ضروری است.

Title: بررسی رابطه سبک مدیریت مربی گری با کیفیت خدمات با استفاده از مدل یابی مسیر به روشPLS در بیمارستانهای آموزشی شهر یزد سال 1391

http://tbj.ssu.ac.ir/article-1-1123-fa.html

مقدمه: بخش بهداشت و درمان، به لحاظ اهمیت نوع خدمات و سر و کار داشتن با سلامت و جان انسانها، اعتلای مدیریت آن و کیفیت خدمات و تضمین آن برای نظام بهداشت و درمان و مردم بطور فزاینده و مداوم مورد توجه قرار گرفته است. این تحقیق به بررسی رابطه سبک مدیریت مربیگری با کیفیت خدمات با استفاده از مدل یابی مسیر به روش حداقل مجذورات جزئی (Partial Least Squares )در بیمارستانهای آموزشی شهر یزد می پردازد. روش بررسی: این مطالعه به روش توصیفی- همبستگی بوده و جامعه آماری شامل کارکنان درمانی وبیماران مراجعه کننده به بیمارستانهای آموزشی شهر یزد است. نمونه آماری از کارکنان300 نفر و بیماران نیز300 نفر و از دو پرسشنامه "سنجش مهارتهای مدیریت مربی گری" و "کیفیت خدمات" برای جمع آوری داده ها استفاده گردید. از مدل یابی معادلات ساختاری روش حداقل مجذورات جزئی و بوسیله نرم افزار Smart PLS برای برآورد پارامترها و بمنظور تجزیه و تحلیل شکاف ادراکات و انتظارات از آزمون t وابسته، تک نمونه ای، مستقل و آزمون تحلیل واریانس یکطرفه با نرم افزار16 spss انجام شد. یافته ها: به کمک مدل PLS، برآورد رابطه ساختاری میان مربی گری و کیفیت خدمات 194/0- به دست آمد. که نشان دهنده رابطه منفی بین مهارتهای مربی گری و کیفیت خدمات است. بنابراین هر چه گرایش به مربی گری در یک بیمارستان بیشتر می شود، ادراک بیماران از کیفیت خدمات در آن بیمارستان کاهش خواهد یافت. نتیجه گیری: به نظر می رسد مدیران ، مدیریت مربی گری و کارهای تیمی در بیمارستانها را بخوبی اجرا ننموده و این ممکن است عللی مانند فرهنگ سازمانی حاکم بر بیمارستان، تفاوت در بخشها ، بالا بودن بیش از حد انتظارات کارکنان و عوامل دیگر داشته باشد. با توجه به مطالب و یافته های این پژوهش به نظر می رسد سبک مدیریت در بیمارستان ، مدیریت استبدادی- خیرخواهانه با محوریت تیم گرایی بوده تا رضایت کارکنان و در نهایت رضایت مشتریان در دریافت بهتر کیفیت خدمات برآورده شود.

Title: مقایسه میزان تاثیر سلامت بر رشد اقتصادی ایران و کشورهای حوزه سند چشم انداز

http://tbj.ssu.ac.ir/article-1-1124-fa.html

مقدمه: خرد هر فرهنگی حکم می کند که " سلامت " ثروت است. زیرا که سلامت یک ملت به عنوان یک عامل تعیین کننده مهم در عملکرد اقتصاد ملی می باشد. اما غالبا سهم هزینه های بهداشتی درمانی از درآمد ناخا لص داخلی در کشورهای توسعه یافته بیشتر از کشورهای کمتر توسعه یافته است. برای مثال این میزان برای اکثر کشورهای OECD بیش از 7درصد و برای ایران حدود 7/5 درصد می باشد. روش بررسی:در این مقاله، رابطه میان برخی از شاخص های وضعیت سلامت (مخارج سلامت، امید به زندگی و نرخ مرگ و میر نوزادان) و تولید ناخالص داخلی واقعی ( (GDPبرای کشورهای حوزه سند چشم انداز در دوره 1995 تا 2010 مورد مطالعه قرار گرفته است. در این راستا از یک الگوی اقتصاد سنجی سلامت ورشد اقتصادی و از آزمون‌های ریشه واحد پنل، پنل هم جمعی وتخمین زننده‌ حداقل مربعات کاملاً اصلاح شده (FMOLS) برای روابط بلند هم جمعی استفاده شده است. یافته ها: یک رابطه بلند مدت بین متغیرها وجود دارد. بین امید به زندگی و تولید ناخالص داخلی واقعی (000/0=P) و همچنین مخارج سلامت و GDPواقعی(000/0=P)یک رابطه مثبت و معنا دار وجود دارد. رابطه بین نرخ مرگ و میر نوزادان و GDP واقعی نیز یک رابطه منفی و معنا دار(000/0=P)می باشد. تمام این نتایج مطابق با تئوری ها می باشد. نتیجه گیری: نتایج نشان می دهد خرج کردن در بازار سلامت وبه تبع آن کسب سلامتی و مطلوبیت اضافه می تواند در افزایش رشد اقتصادی مؤثر باشد. وضعیت سلامت در ایران نیز بر رشد اقتصادی تاثیری مثبت دارد، اما در مقام مقایسه، ایران در بین کشورهایی است که با مخارج بیشتر نتایج ضعیف تری بدست آورده است. این خود می تواند انگیزه ای برای سیاست گذاران ایجاد کند تا در کشورهای حوزه سند چشم انداز به مقوله سلامت اهمیت بیشتری داده شود. اما باز هم سوالاتی در مورد نحوه تخصیص منابع و کارایی تخصیصی باقی می ماند.

Title: بررسی کارایی حذف جیوه با استفاده از نانوذرات هیبریدی TiO2/SiO2از محیط های آبی

http://tbj.ssu.ac.ir/article-1-1125-fa.html

مقدمه: جیوه یک ترکیب سمی است. تجمع بیولوژیکی جیوه بخصوص در آبزیان مشکلات بهداشتی زیادی بوجودآورده است. روش‌های مختلفی جهت حذف فلزات سنگین از پساب های صنعتی بکار رفته است. جذب سطحی یکی از روش‌های فیزیکی شیمیایی است که بعلت بازدهی بالا، سادگی ودر دسترس بودن انواع مختلف آنها روشی پرکاربرد است. این تحقیق با هدف کارائی نانوذرات هیبریدیTiO2/SiO2 در حذف جیوه از فاضلاب صنعتی انجام گرفت. روش بررسی: مطالعه -تجربی است که در فاصله زمانی مهر لغایت اسفند1391 انجام گرفت تحقیق هم زمان برروی نمونه سینتیک ونمونه واقعی فاضلاب رنگ سازی انجام شد. پس از تعیین کیفیت شیمیایی فاضلاب خام، محلول سنتتیک تهیه و اثر pH(9،5،7،3)، مقدارجاذب (1 و 75/0 ،5/0 ،25/0 گرم بر لیتر)، زمان های تماس(30،60،90،120 دقیقه ) و غلظت های مختلف جیوه(20،30،50،70 و90 میلی گرم بر لیتر) بر روی میزان حذف بررسی و شرایط بهینه هر یک از پارامترها تعیین گردید. به منظور درک بهتر فرایند جذب، سینتیک جذب و ایزوترم های تعادلی بررسی شد. برای تجزیه تحلیل داده ها از نرم افزار SPSSنسخه 16 و آزمون رگرسیون، ضریب پیرسون و درجه معنی داری استفاده شد. یافته ها: بیشترین راندمان حذف جیوه در 5=pH، مقدارجاذب 5/0گرم بر لیتر و زمان ماند 30 دقیقه برای محلولهای سنتتیک و واقعی به ترتیب 86/99%و 56/95% بدست آمد و غلظت جیوه 50 میلی گرم بر لیتر بالاترین درصد حذف86/99% را نتیجه داد. نتایج نشان داد، جذب جیوه ازایزوترم فروندلیچ با ضریب همبستگی)56/0 (R2 =و سنتتیک واکنش نوع اول با ضریب همبستگی)63/0 (R2 = پیروی می کند. نتیجه گیری: با توجه به درصد بالای حذف جیوه در شرایط بهینه توسط نانوذره هیبریدی و مقایسه آن با روش های دیگر می توان روش مذکور را بعنوان یکی از روش های کارآمد برای حذف جیوه مطرح نمود.

Title: بررسی حذف کروم از آب با استفاده از جاذب‌های پوست موز، پوست هندوانه، خاک اره و نانو ذرات اکسید آهن

http://tbj.ssu.ac.ir/article-1-1126-fa.html

مقدمه: در تحقیقات زیادی از نانوذرات مختلف برای حذف فلزات سنگین از آب استفاده شده‌اند، نتایج این تحقیقات نشان دهنده توان بالای این مواد برای حذف مواد آلاینده از محلول‌های آبی هستند همچنین جاذب‌های زیستی نیز دارای توان زیادی برای تصفیه آب می‌باشند. هدف اصلی این تحقیق، بررسی تاثیرات اضافه کردن نانوذرات اکسید آهن به فیلتر‌های زیستی حذف کروم از آب است. روش بررسی: در این پژوهش تجربی فرآیند مورد استفاده فیلتراسیون بود، همچنین در این تحقیق متغیر مورد بررسی در فرآیند نوع جاذب بود در واقع تاثیر تغییر نوع جاذب بر درصد حذف فلز سنگین مورد بررسی قرار گرفت، از سوی دیگر روش کار نیز به این شکل بود که در دو گام اول با استفاده از جاذب-های خاک و خاک اره، آب آلوده با سه غلظت متفاوت 7/883، 4/1767 و ppm 8837 پالایش گردید در دو گام بعدی از پژوهش نیز برای مقایسه به هر کدام از جاذب‌های خاک و خاک اره مقادیری از مخلوط نانوذرات اکسید آهن با چند جاذب گیاهی دیگر اضافه شد، و دوباره جذب در همین غلظت‌های اولیه انجام گرفت. یافته‌ها: نتایج نشان داد که بهترین جاذب، جاذب خاک و نانوذرات اکسید آهن با حداکثر درصد حذف معادل 2/96 است، همچنین بهترین عملکرد در غلظت اولیه فلز سنگین ppm 8837 بدست آمد، از سوی دیگر در آزمایش‌هائی که از نانوذرات اکسید آهن استفاده شد، اضافه شدن نانوذرات باعث افزایش میزان جذب کروم و تبدیل Cr6+ به Cr3+ گردید. نتیجه گیری: با استفاده از نتایج این تحقیق می‌توان گفت که ترکیب شدن نانوذرات اکسید آهن با مواد فیلترهای حذف کروم می‌تواند Cr6+ را به Cr3+ تبدیل کند همچنین این کار بازده فرآیند حذف را نیز افزایش می‌دهد.

Title: فعالیت ضد قارچی عصاره تام آبی سرشاخه های هوایی گیاه انغوزه(ferula assa foetida) بر رشد کاندیدا آلبیکنس و مقایسه ان با فلوکونازول در شرایط برون تنی

http://tbj.ssu.ac.ir/article-1-1127-fa.html

مقدمه:کاندیدیازیس از شایعترین بیماریهای قارچی انسانی است که با توجه به افزایش روز افزون جمعیت ایمنوساپرس، دیابتی و سرطانی، روز به روز بر میزان شیوع این بیماری و مقاومت دارویی عوامل بیماری آن افزوده می شود. کاندیدا آلبیکنس از قارچهای مخمری فلور نرمال بدن انسانی بعنوان شایعترین عامل این بیماری شناخته می شود. هدف از انجام مطالعه حاضرارزیابی خواص ضد کاندیدایی عصاره تام آبی سرشاخه های هوایی گیاه انغوزه و مقایسه آن با فلوکونازول در شرایط In vitro است. روش بررسی:در این مطالعه تجربی ابتدا عصاره تام آبی mg/ml 35 از سرشاخه های هوایی گیاه ا نغوزه بومی ایران تهیه و سپس با کمک روش Broth microdilution ، میزان حساسیت دارویی سوش استانداردکاندیدا آلبیکنس نسبت به غلظتهای مختلف این عصاره درشرایط برون تنی بررسی و مقادیر حداقل غلظت مهار کنندگی و کشندگی این عصاره و داروی فلوکونازول برروی سوسپانسیون کاندیدا محاسبه گردید. یافته ها:غلظتmg/ml 75/8 از عصاره سرشا خه های هوایی انغوزه توانست بطور کامل از رشد کاندیدا آلبیکنس جلوگیری کرده و تمامی سلولهای زنده کاندیدا در این غلظت را از بین ببرد (MFC). بعلاوه غلظت های 273/0وmg/ml 4/4 عصاره آبی انغوزه به ترتیب به عنوان حداقل مقادیر مهارکنندگی رشد 50% و 90% کاندیدا بدست آمد. در رابطه با فلوکونازول غلظتµg/ml 128 به عنوان MFC و غلظت µg/ml5/0 به عنوان MIC50 بدست آمد. نتیجه گیری: نتایج مطالعه حاضر نشان می دهد که عصاره تام آبی برگ و سرشاخه های هوایی گیاه انغوزه اثر مهاری و کشندگی بر علیه قارچ کاندیدا آلبیکنس در شرایط برون تنی داشته و پیشنهاد می شود مطالعات بیشتر در شرایط درون تنی نیز انجام شود.

Title: بررسی نرم قد، وزن و نمایه توده بدنی ( BMI) در کودکان 11-6 سال شهر یزد در سال 90-1389

http://tbj.ssu.ac.ir/article-1-1128-fa.html

مقدمه: ارزیابی روند رشد بویژه در اطفال از اهمیت خاصی برخوردار است. برای این کار لزوما باید از داده های استاندارد مربوطه استفاده شود. با توجه به تفاوت های ژنتیکی و الگوی زیستی مردم مناطق مختلف، بهتر است به جای استفاده از استاندارد های غیر بومی از موارد بومی یا ملی استفاده شود. هدف از این مطالعه بومی کردن داده های نرمال قد، وزن و نمایه توده بدنی کودکان 11-6 ساله در شهر یزد بوده است. روش بررسی: این مطالعه به صورت توصیفی از نوع مقطعی در سال تحصیلی1390- 1389 در سطح شهر یزد انجام شد و 26 مدرسه به روش نمونه گیری خوشه ای انتخاب و مشخصات تمام دانش اموزان یک کلاس از هر پایه اندازه گیری شد. قد و وزن 216 کودک در گروه های سنی 6 ،7 ،8... ، 11 سال ( جمعا 2592 کودک) اندازه گیری گردید. با استفاده از نرم افزار آماری16 SPSS منحنی های قد و وزن و نمایه توده بدنی بر اساس سن و جنس محاسبه شده و با استاندارد جهانی CDC مورد مقایسه قرار گرفتند. یافته ها: منحنی های صدک های قد برای سن کودکان دختر زیر منحنی های استاندارد CDC بودند. منحنی مربوط به صدک های۵ و ۱۵ وزن برای سن در پسران در اکثر گروه های سنی زیر منحنی استاندارد بود. منحنی مربوط به صدک های 95 و 97 توده بدنی کودکان دختر و پسر در سنین بالای 10 سال بالاتر از استاندارد بود. نتیجه گیری: با توجه به تفاوت های مشاهده شده بین منحنی های قد، وزن و نمایه توده بدنی کودکان یزدی و استاندارد CDC، بهتر است از نرم های این مطالعه برای کودکان 11-6 ساله یزدی استفاده شود.
